# In Silico investigation of Danshen (*Salvia miltiorrhiza*) derivatives as potential inhibitor of amyloid beta fibril and design of promising lead compounds as Anti Alzheimer’s drug by pharmacophore modeling

**DOI:** 10.1002/alz70859_103299

**Published:** 2025-12-25

**Authors:** Lutful Haque Saran

**Affiliations:** ^1^ Gono University, Dhaka Bangladesh

## Abstract

**Background:**

Danshen (*Salvia miltiorrhiza*) is a kind of rhizome and traditionally being used for the long time in China. In recent years some exceptional experimental works have been done on Danshen derivatives and evidence have been found that several Danshen derivatives are able to inhibit amyloid beta fibril, a pathological hallmark of Alzheimer’s disease. Six molecules named Salvianolic acid A, Cryptotanshinone, Dihydro Tanshinone, Rosmarinic acid, Tanshinone I and Tanshinone II have been identified from different literature, as all the molecules have been shown significant inhibition to the amyloid beta fibril so they must contain a common structure which is useful for pharmacophore modeling.

**Method:**

Six molecular structures were collected from PubChem and analyzed in PharmaGist for pharmacophore parameter searching, followed by pharmacophore model development using PyMOL. The ZINC database was screened using the pharmacophore model, yielding 3328 potential molecules, which were further filtered through the RPBS Mobyle web server for druggability, resulting in 1042 molecules for further study. These 1042 molecules, along with the six parent molecules, were docked into the active sites of amyloid beta fibril (PDB ID: 2MXU), identified via literature and web servers, with 39 molecules showing better binding affinity than the parent compounds. The selected molecules underwent ADME, Lipinski’s rule, and drug‐likeness analyses for further evaluation.

**Result:**

Among the 39 top ranked molecules 6 molecules have been rejected based on Lipinski’s violation and finally 33 molecules have been selected as promising lead compounds. As these lead compounds have shown better binding affinity than the experimentally tested molecules, they have enough potential to show inhibitory action against amyloid beta fibril. All these molecules have been subjected to molecular dynamics simulation for testing the stability of binding affinity and the dynamics of these interactions with amyloid beta fibril. The molecular dynamics simulation process is still ongoing.

**Conclusion:**

Significant evidence for the effectiveness of the In‐silico screened molecules against amyloid beta fibril has been presented in the current study; nonetheless, further experimental studies are needed to assess the potential of the compounds as possible drugs against Alzheimer’s disease.

